# Blood pressure indices for predicting incident cardiovascular disease: A 13-year follow-up study in Japanese population

**DOI:** 10.1016/j.ajpc.2025.101341

**Published:** 2025-11-03

**Authors:** Takahiro Ichikawa, Hiroshi Okada, Hanako Nakajima, Emi Ushigome, Masahide Hamaguchi, Kazushiro Kurogi, Hiroaki Murata, Eri Tsuda, Naoki Yoshida, Masato Ito, Michiaki Fukui

**Affiliations:** a Department of Endocrinology and Metabolism, Kyoto Prefectural University of Medicine, Graduate School of Medical Science, 465 Kajii-cho, Kawaramachi-Hirokoji, Kamigyo-ku, Kyoto 602-8566, Japan; b Department of Diabetes and Endocrinology, Matsushita Memorial Hospital, 5-55 Sotojima-cho, Moriguchi 570-8540, Japan; c Department of Health Care Center, Panasonic Health Insurance Organization, 5-55 Sotojima-cho, Moriguchi 570-8540, Japan; d Department of Orthopaedic Surgery, Matsushita Memorial Hospital, 5-55 Sotojima-cho, Moriguchi 570-8540, Japan

**Keywords:** Blood pressure indices, Cardiovascular events, Japan

## Abstract

**Background:**

Several blood pressure (BP) indices have been associated with incident cardiovascular disease (CVD); however, evidence comparing their long-term prognostic value in Asian populations is limited. We investigated the association between multiple BP indices and CVD risk over a 13-year follow-up period in a large Japanese population.

**Methods:**

Data from a health check-up program conducted by the Panasonic Corporation covering 166 operational sites from 2008 to 2021, including 163,956 participants not receiving anti-hypertensive drugs, were analyzed. The primary outcome was the incidence of three-point major adverse cardiac events (MACE), including cardiovascular death, nonfatal coronary artery disease, and nonfatal stroke. Cox proportional hazards models and time-dependent receiver operating characteristic (ROC) analyses were used to evaluate the associations between the four BP indices (systolic BP [SBP], diastolic BP [DBP], pulse pressure [PP], and mean arterial pressure [MAP]) and incident MACE.

**Results:**

After adjusting for confounders, all four BP indices were found to be independently associated with incident MACE. Among them, MAP demonstrated the highest area under the ROC curve for predicting MACE. In the gender-stratified analyses, the findings in males were broadly consistent with the overall results, whereas the predictive advantage of MAP was attenuated in females. Similarly, in analyses restricted to participants aged ≥50 years, the superiority of the MAP was less evident.

**Conclusions:**

MAP was the strongest predictor of incident CVD in the Japanese population. These findings underscore the importance of BP phenotyping and suggest that gender and age may modify the utility of MAP in cardiovascular risk stratification.

## 1 Introduction

The global prevalence of hypertension has been increasing, with an estimated 1.39 billion adults worldwide reported to have hypertension in 2010 [1] Hypertension is associated with an increased risk of cardiovascular disease (CVD) and mortality [2,3] Hypertension guidelines recommend reducing blood pressure to below 140/90 mmHg as the primary treatment goal [4] Mills et al. [1] highlighted that despite the availability of effective lifestyle modifications and pharmaceutical treatments, the levels of awareness, treatment, and control of hypertension remain inadequate, particularly in low-income and middle-income countries. Therefore, hypertension remains a persistent global public health challenge.

Blood pressure (BP) indices included systolic BP (SBP), diastolic BP (DBP), pulse pressure (PP), and mean arterial pressure (MAP). PP is defined as the difference between SBP and DBP, and represents the fluctuation between peak and trough pressures during a cardiac cycle [5] MAP represents the average pressure over a cardiac cycle and reflects both the cardiac output and systemic vascular resistance [6,7] Blood pressure can be conceptualized as having two key hemodynamic components: a stable component, represented by MAP, and a pulsatile component, represented by PP [8] While the pulsatile component is increasingly recognized as a key contributor to end-organ damage [9] particularly in the brain, [10] the relative prognostic importance of these two components for cardiovascular disease may differ depending on the characteristics of the population, such as age [8]

Accumulating evidence has highlighted the association between BP measurements, including SBP, DBP, and PP, and CVD risk [11] Additionally, Zheng et al. [12] reported that the MAP is associated with the risk of ischemic stroke. Several studies that have compared the predictive abilities of different BP indices for CVD in patients with diabetes or a history of coronary artery disease (CAD), [13,14] have presented inconsistent results. Moreover, some investigations have assessed the prognostic performance of these indices in untreated individuals, [15,18] although reports from Asian populations remain scarce, [17,18] and follow-up periods have generally been limited.

Therefore, we aimed to compare the associations between SBP, DBP, MAP, and PP and the incidence of CVD during a 13-year follow-up period in a Japanese population.

## 2 Materials and methods

### 2.1 Study participants and design

This longitudinal cohort study was conducted among employees of the Panasonic Corporation in Osaka, Japan, as part of an annual corporate health-screening program. This program was designed to promote public health by facilitating the early detection of chronic diseases, including metabolic disorders, through regular risk factor assessments. According to Japanese Occupational Health and Safety Law, annual health checkups are mandatory for all employees. We used data from the Panasonic Cohort Study, comprising health examination records collected between 2008 and 2021. The study protocol was approved by the local ethics committee of the Panasonic Health Insurance Organization (approval number: 2021–001) and the study was conducted in accordance with the principles of the Declaration of Helsinki. Written informed consent was not obtained because the study employed an opt-out approach consistent with the ethical guidelines of the Ministry of Health, Labour, and Welfare in Japan.

### 2.2 Participants

**Fig. 1:**
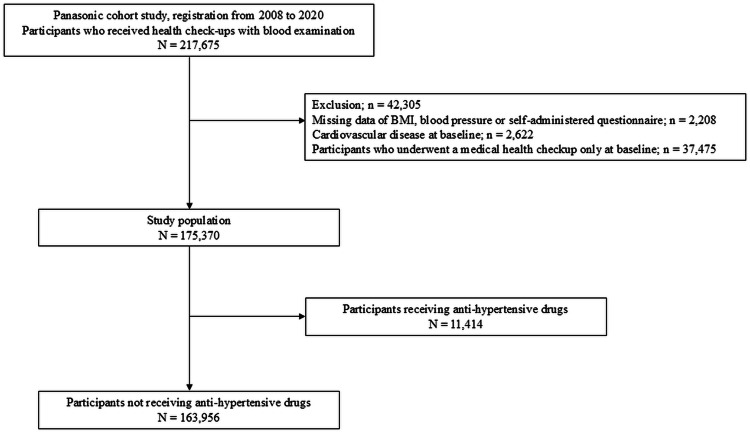
A flow diagram of the selection of participants.

Eligible participants were individuals aged ≥18 years who had undergone annual health examinations with blood testing between 2008 and 2021. Baseline was defined as the date of the earliest available health examination within this period. Participants were excluded if they met any of the following criteria: (1) only attended the baseline examination without subsequent follow-up, often due to retirement; (2) had missing data on key variables such as body mass index (BMI), BP, or self-administered questionnaire; or (3) had a known history of CAD or stroke at baseline. The participant selection process is illustrated in Fig. 1.

### 2.3 Definition of blood pressure indices

Blood pressure was measured once with a validated automated sphygmomanometer after participants had rested in a seated position for a minimum of several minutes. We compared four distinct BP indices: SBP, DBP, PP, and MAP. MAP and PP are composite measures derived from SBP and DBP, and were calculated using the following formulas [19]:

PP=SBP−DBP

$$MAP=\frac{SBP-DBP}{3}\;+DBP$$

### 2.4 Definition of cardiovascular events and outcomes

Information on cardiovascular events and related mortality was obtained from the Panasonic Cohort Study Database. The incidence of cardiovascular events in 2021 was assessed. CAD was defined as the occurrence of either angina pectoris or acute myocardial infarction. Stroke was defined as a cerebral infarction or intracerebral hemorrhage. Cardiovascular death was defined as death attributable to CAD or stroke. The primary outcome was the occurrence of three-point major adverse cardiovascular events (MACE), comprising cardiovascular death, nonfatal CAD, and nonfatal stroke. The secondary outcomes were CAD and stroke. All causes of death, including cardiovascular death, were identified based on the diagnostic codes recorded in the healthcare data. Information regarding the medical history of CAD and stroke of participants was collected using a self-administered questionnaire.

### 2.5 Definitions of impaired fasting glucose, hypertension, and dyslipidemia

Dyslipidemia was defined as either low-density lipoprotein (LDL) cholesterol, high-density lipoprotein (HDL) cholesterol, or triglyceride levels of ≥140 mg/dL, ≤40 mg/dL, or ≥150 mg/dL, respectively, [20] or as the current use of lipid-lowering medications. Impaired fasting glucose was diagnosed based on fasting blood glucose levels of ≥110 mg/dL [21] and on the current use of glucose-lowering medications.

### 2.6 Covariables

All biochemical measurements were performed according to standardized laboratory protocols. Venous blood samples were collected in the morning following an overnight fast of at least 10 h. Baseline information on lifestyle factors was obtained using a previously validated self-administered questionnaire. Smoking status was classified as none, past, or current. Regular physical activity was defined as participation in any form of exercise at least twice per week. Alcohol consumption was assessed by both frequency and volume of intake. Daily ethanol intake (g/day) was calculated, and individuals consuming ≥20 g/day were classified as alcohol drinkers, consistent with the “moderate drinking” criterion established by the Ministry of Health, Labour and Welfare of Japan [22]

### 2.7 Statistical analyses

The analysis was restricted to participants who did not receive antihypertensive medications. Associations between SBP, DBP, MAP, and PP, and incident MACE, stroke, and CAD were evaluated using Cox proportional hazards models. In multivariate analyses, model 1 was adjusted for gender, age, BMI, alcohol consumption (Daily ethanol intake ≥20 g/day or <20 g/day), smoking status (none, past, current), and physical activity (yes or no), as defined in the Section 2.6. Model 2 was adjusted for impaired fasting glucose (IFG) and dyslipidemia, as defined in the Section 2.5. To further assess the incremental predictive value of each BP index, likelihood ratio tests were performed by sequentially adding each index to a base model that included the eight covariates specified in Model 2.

To assess the predictive ability of each BP index, we constructed time-dependent receiver operating characteristic (ROC) curves for censored survival data and calculated the area under the curve (AUC). The optimal cut-off values for SBP, DBP, MAP, and PP were identified for predicting the RISK of MACE, stroke, and CAD. The AUCs for the four BP indices were compared using bootstrap resampling. We also conducted subgroup analyses stratified by gender to evaluate whether the associations between BP indices and cardiovascular outcomes differed between males and females.

All continuous variables are presented as mean ± standard deviation, and categorical variables are presented as counts. Statistical significance was defined as a two-sided p-value of < 0.05.

In the sensitivity analysis, we included all participants, regardless of their antihypertensive medication status, to evaluate the robustness of the findings. Additionally, to further evaluate the robustness of our findings, we conducted a sensitivity analysis limited to participants aged ≥50 years who were not receiving antihypertensive therapy. All statistical analyses were conducted using JMP v.17 (SAS Institute, Cary, NC, USA) except for the time-dependent ROC curve analysis, which was performed using the "survival ROC" package in R v.4.3.2 (R Foundation for Statistical Computing, Vienna, Austria).

## 3 Results

A total of 175,370 participants were included in this study. The primary analysis was restricted to 163,956 individuals who did not take antihypertensive medication at baseline. The baseline characteristics of the study population are shown in Table 1, and those of the individuals taking and not taking anti-hypertensive drugs are presented in Table S1. Among the 175,370 participants, 4519 (2.6 %) developed MACE, 1534 (0.9 %) developed stroke, and 3052 (1.7 %) developed CAD. Among those not receiving antihypertensive treatment, 3694 (2.3 %), 1253 (0.8 %), and 2479 (1.5 %) developed MACE, stroke, and CAD, respectively. The adjusted hazard ratios (HRs) of the covariates for incident MACE, stroke, and CAD among the participants not receiving anti-hypertensive drugs are shown in Table 2.

**Table 1:** Baseline characteristics of participants in the primary analysis not receiving antihypertensive medications.

	Anti-hypertensive drugs (-)
N	163,956
Age (y)	42.8 (9.0)
Gender (males/females) ( %)	118,753/45,203 (72.4/27.6)
Body mass index (kg/m^2^)	22.8 (3.4)
Systolic blood pressure (mmHg)	117.7 (14.4)
Diastolic blood pressure (mmHg)	73.1 (10.9)
Pulse pressure (mmHg)	44.6 (8.8)
Mean arterial pressure (mmHg)	88.0 (11.5)
Triglycerides (mg/dL)	107.3 (88.5)
HDL cholesterol (mg/dL)	60.9 (15.3)
LDL cholesterol (mg/dL)	122.0 (31.7)
Fasting plasma glucose (mg/dl)	93.5 (16.3)
Glucose-lowering medications (±)	2306/161,650 (1.4/98.6)
Lipid-lowering medications (±)	3437/160,519 (2.1/97.9)
Smoking (none/past/current)( %)	91,224/21,033/51,699 (55.6/12.8/31.5)
Drinker (±) ( %)	36,030/127,926 (22.0/78.0)
Physical exercise (±) ( %)	28,084/135,872 (17.1/82.9)

Data are presented as mean (standard deviation, or percentage) or absolute number.

Abbreviations: LDL, low-density lipoprotein; HDL, high-density lipoprotein.

**Table 2:** Unadjusted hazard ratios and multivariate adjusted hazard ratios for MACE, stroke, CAD in participants not receiving antihypertensive medications. (per SD).

MACE	Crude		Model 1		Model 2	
	Hazard ratios (95 % CI) C-Statistics (95 % CI)	*p*	Hazard ratios (95 % CI) C-Statistics (95 % CI)	*p*	Hazard ratios (95 % CI) C-Statistics (95 % CI)	*p*
SBP	1.50 (1.46–1.55) 0.623 (0.605–0.641)	<0.001	1.30 (1.26–1.35) 0.686 (0.671–0.701)	<0.001	1.28 (1.24–1.32) 0.691 (0.675–0.706)	<0.001
DBP	1.56 (1.52–1.61) 0.637 (0.619–0.656)	<0.001	1.34 (1.29–1.38) 0.688 (0.672–0.703)	<0.001	1.31 (1.27–1.36) 0.692 (0.677–0.708)	<0.001
PP	1.14 (1.11–1.18) 0.529 (0.510–0.548)	<0.001	1.10 (1.07–1.14) 0.675 (0.660–0.690)	<0.001	1.09 (1.06–1.13) 0.681 (0.666–0.696)	<0.001
MAP	1.56 (1.52–1.61) 0.639 (0.621–0.657)	<0.001	1.35 (1.30–1.39) 0.689 (0.673–0.704)	<0.001	1.32 (1.28–1.37) 0.693 (0.678–0.708)	<0.001
Stroke	Crude		Model 1		Model 2	
	Hazard ratios (95 % CI) C-Statistics (95 % CI)	*p*	Hazard ratios (95 % CI) C-Statistics (95 % CI)	*p*	Hazard ratios (95 % CI) C-Statistics (95 % CI)	*p*
SBP	1.56 (1.48–1.64) 0.633 (0.601–0.664)	<0.001	1.38 (1.31–1.46) 0.676 (0.649–0.702)	<0.001	1.37 (1.30–1.45) 0.678 (0.651–0.704)	<0.001
DBP	1.66 (1.57–1.74) 0.655 (0.624–0.686)	<0.001	1.46 (1.38–1.55) 0.682 (0.656–0.709)	<0.001	1.45 (1.37–1.53) 0.684 (0.658–0.711)	<0.001
PP	1.14 (1.07–1.20) 0.527 (0.495–0.559)	<0.001	1.10 (1.04–1.16) 0.656 (0.630–0.682)	0.001	1.09 (1.03–1.15) 0.659 (0.633–0.685)	0.002
MAP	1.64 (1.56–1.72) 0.654 (0.623–0.685)	<0.001	1.46 (1.38–1.54) 0.682 (0.656–0.709)	<0.001	1.44 (1.36–1.53) 0.684 (0.658–0.711)	<0.001
CAD	Crude		Model 1		Model 2	
	Hazard ratios (95 % CI) C-Statistics (95 % CI)	*p*	Hazard ratios (95 % CI) C-Statistics (95 % CI)	*p*	Hazard ratios (95 % CI) C-Statistics (95 % CI)	*p*
SBP	1.46 (1.41–1.51) 0.616 (0.594–0.638)	<0.001	1.25 (1.20–1.30) 0.686 (0.667–0.704)	<0.001	1.22 (1.18–1.27) 0.692 (0.674–0.711)	<0.0001
DBP	1.49 (1.44–1.55) 0.624 (0.602–0.646)	<0.001	1.25 (1.20–1.30) 0.685 (0.666–0.704)	<0.001	1.23 (1.17–1.28) 0.692 (0.673–0.710)	<0.001
PP	1.15 (1.11–1.20) 0.531 (0.508–0.554)	<0.001	1.11 (1.06–1.15) 0.679 (0.660–0.697)	<0.001	1.10 (1.05–1.14) 0.687 (0.668–0.705)	<0.001
MAP	1.50 (1.45–1.56) 0.628 (0.606–0.650)	<0.001	1.27 (1.22–1.32) 0.686 (0.668–0.705)	<0.001	1.24 (1.19–1.29) 0.693 (0.674–0.711)	<0.001

Data are presented as hazard ratios (95 % confidence intervals).

Model 1 was adjusted for gender, age, BMI, drinking habits, smoking status, and physical exercise.

Model 2 was adjusted for gender, age, BMI, drinking habits, smoking status, physical exercise, the presence of IFG, and the presence of dyslipidemia.

Abbreviations: SD, standard deviation; CI, confidence intervals; SBP, systolic blood pressure; DBP, diastolic blood pressure; PP, pulse pressure; MAP, mean arterial pressure; MACE, major adverse cardiovascular events; CAD, coronary artery disease; BMI, Body mass index; IFG, impaired fasting glucose.

**Fig. 2:**
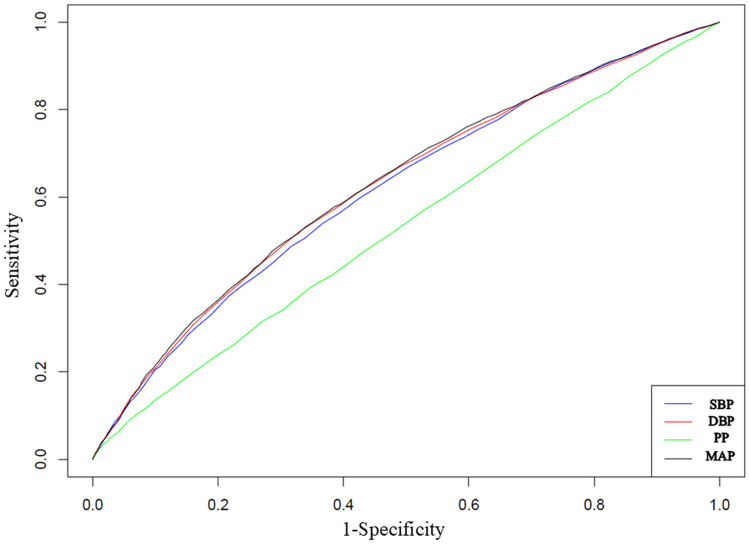
Receiver operating characteristic curve showing the ability of SBP, DBP, PP, and MAP to determine the incidence of MACE after 13 years of follow-up in participants who did not receive antihypertensive medications. Abbreviations: SBP, systolic blood pressure; DBP, diastolic blood pressure; PP, pulse pressure; MAP, mean arterial pressure; MACE, major adverse cardiovascular events.

Multivariate analysis revealed significant associations between SBP (HR: 1.28 per SD; 95 % CI: 1.24–1.32), DBP (HR: 1.31 per SD; 95 % CI: 1.27–1.36), PP (HR: 1.09 per SD; 95 % CI: 1.06–1.13), and MAP (HR: 1.32 per SD; 95 % CI: 1.28–1.37), and the risk of incident MACE. The strength and direction of the associations were similar between the stroke and CAD groups. Likelihood ratio tests were performed to evaluate the predictive utility of each BP index in participants not receiving antihypertensive medication. The inclusion of these indices significantly improved the model performance in participants not receiving antihypertensive medications in predicting risk of MACE, stroke, and CAD (Tables S2–S4). Table 3 presents the AUC values and optimal cut-off points at 13 years derived from the time-dependent ROC curve analysis in participants not receiving antihypertensive treatment. For MACEs, the AUC values for SBP, DBP, PP, and MAP were 0.618, 0.625, 0.532, and 0.629, respectively, and the corresponding cut-off values were 121, 77, 47, and 91.7. Comparable trends were observed for stroke and CAD (Table 3). Table 4 shows the results of comparisons of the AUC values of SBP, DBP, PP, and MAP for MACE in participants who did not receive antihypertensive treatment. MAP demonstrated superior predictive ability than SBP, DBP, and PP. Additionally, a similar pattern was observed for CAD, wherein MAP exhibited superior discriminative capacity, consistent with the findings for MACE. MAP also demonstrated a greater predictive performance for stroke risk than SBP and PP. Fig. 2 shows the time-dependent ROC curves for SBP, DBP, PP, and MAP in relation to MACE. Figures S1 and S2 illustrate the corresponding curves for stroke and CAD, respectively.

**Table 3:** The area under the curve and optimal cut-off values with incident MACE, stroke, and CAD in participants not receiving antihypertensive medications.

**MACE**								
	AUC (95 % CI)	Cut-off value	Sensitivity	Specificity	PPV	NPV	PLR	NLR
SBP	0.618 (0.607–0.627)	121	54.1 %	63.2 %	6.7 %	96.6 %	1.470	0.727
DBP	0.625 (0.615–0.633)	77	52.1 %	67.1 %	7.2 %	96.6 %	1.580	0.715
PP	0.532 (0.523–0.541)	47	39.4 %	65.2 %	5.3 %	95.6 %	1.132	0.930
MAP	0.629 (0.619–0.637)	91.7	53.3 %	65.9 %	7.1 %	96.6 %	1.565	0.708
**Stroke**								
	AUC (95 % CI)	Cut-off value	Sensitivity	Specificity	PPV	NPV	PLR	NLR
SBP	0.630 (0.612–0.647)	123	51.9 %	67.8 %	2.5 %	98.9 %	1.612	0.709
DBP	0.647 (0.631–0.663)	78	52.8 %	70.1 %	2.7 %	98.9 %	1.765	0.673
PP	0.530 (0.511–0.548)	39	76.3 %	28.6 %	1.7 %	98.7 %	1.069	0.828
MAP	0.648 (0.632–0.664)	91.3	59.1 %	6.4 %	2.6 %	99.0 %	1.661	0.635
**CAD**								
	AUC (95 % CI)	Cut-off value	Sensitivity	Specificity	PPV	NPV	PLR	NLR
SBP	0.610 (0.599–0.622)	121	53.6 %	63.0 %	4.8 %	97.5 %	1.447	0.737
DBP	0.610 (0.599–0.622)	77	50.2 %	66.7 %	5.0 %	97.5 %	1.509	0.746
PP	0.535 (0.522–0.546)	47	40.4 %	65.2 %	3.9 %	96.9 %	1.159	0.915
MAP	0.616 (0.606–0.629)	89.3	59.3 %	57.8 %	4.7 %	97.6 %	1.405	0.704

Abbreviations: MACE, major adverse cardiovascular event; CAD, coronary artery disease; AUC, area under the curve; PPV, positive predictive value; NPV, negative predictive value; PLR, positive likelihood ratio; NLR, negative likelihood ratio.

**Table 4:** Comparison of area under the curve of SBP, DBP, PP, and MAP with incident MACE, stroke, and CAD in participants not receiving antihypertensive medications.

**MACE**	vs. SBP		vs. DBP		vs. PP	
	difference value	95 % CI P value	difference value	95 % CI P value	difference value	95 % CI P value
SBP	reference		-		-	
DBP	0.008	0.001 to 0.013 *P* = 0.028	reference		-	
PP	−0.084	−0.094 to −0.076 *P* < 0.001	−0.092	−0.105 to −0.079 *P* < 0.001	reference	
MAP	0.012	0.007 to 0.015 *P* < 0.001	0.004	0.002 to 0.006 *P* < 0.001	0.096	0.085 to 0.108 *P* < 0.001
**Stroke**	vs. SBP		vs. DBP		vs. PP	
	difference value	95 % CI P value	difference value	95 % CI P value	difference value	95 % CI P value
SBP	reference		-		-	
DBP	0.018	0.005 to 0.029 *P* = 0.005	reference		-	
PP	−0.101	−0.114 to −0.084 *P* < 0.001	−0.119	−0.139 to −0.092 *P* < 0.001	reference	
MAP	0.019	0.011 to 0.026 *P* < 0.001	0.001	−0.003 to 0.006 *P* = 0.535	0.121	0.095 to 0.138 *P* < 0.001
**CAD**	vs. SBP		vs. DBP		vs. PP	
	difference value	95 % CI P value	difference value	95 % CI P value	difference value	95 % CI P value
SBP	reference		-		-	
DBP	0.000	−0.009 to 0.009 *P* = 0.992	reference		-	
PP	−0.077	−0.087 to −0.064 *P* < 0.001	−0.078	−0.094 to −0.058 *P* < 0.001	reference	
MAP	0.007	0.001 to 0.012 *P* = 0.035	0.007	0.003 to 0.009 *P* = 0.001	0.084	0.067 to 0.098 *P* < 0.001

Abbreviations: MACE, major adverse cardiovascular event; CAD, coronary arterial disease; SBP, systolic blood pressure; DBP, diastolic blood pressure; PP, pulse pressure; MAP, mean arterial pressure.

In addition to the primary analyses, subgroup analyses stratified by gender were performed (Tables S5 and S6). Although results in males were broadly consistent with the main findings, analyses results in females showed a different pattern, suggesting potential gender-related differences in the predictive value of BP indices (Table S6).

Sensitivity analysis including all participants, regardless of antihypertensive medication use, confirmed that the associations between each BP index and MACE remained consistent with those observed in the primary analysis (Table S7). In the sensitivity analysis incorporating all participants, the AUC values and corresponding optimal cut-off points for SBP, DBP, PP, and MAP at 13 years were comparable with those observed in the primary analysis (Table S8). Notably, MAP consistently exhibited superior discriminative ability for predicting MACE compared with SBP, DBP, and PP, thereby reinforcing the robustness of the main findings (Table S9). Figure S3 displays the time-dependent ROC curves for SBP, DBP, PP, and MAP in relation to MACE among the entire cohort, including patients receiving antihypertensive medications. Figures S4 and S5 illustrate the corresponding curves for stroke and CAD, respectively. To further evaluate the robustness of our findings, we conducted a sensitivity analysis restricted to participants aged >50 years who were not receiving antihypertensive therapy. The HRs for MACE, stroke, and CAD per SD increase were broadly consistent with those in the primary analysis (Table S10). In this subgroup, MAP demonstrated superior discriminative performance for predicting MACE compared with PP and DBP, whereas its predictive ability did not exceed that of SBP (Table S11).

## 4 Discussion

This study evaluated the association between multiple BP indices and CVD risk over a 13-year follow-up period in a large Japanese population. The main findings were as follows: (1) all BP indices were associated with MACE, stroke, and CAD, and (2) MAP was more predictive of incident MACE than SBP, DBP, and PP within 13 years. (3) A cutoff value of 91.7 mmHg for MAP was determined to predict the future incidence of MACE within 13 years; therefore, MAP might be most useful in predicting incident CVD within 13 years in a Japanese population. However, the prognostic advantage of MAP was less evident in females and participants aged > 50 years, indicating that gender and age may modify the utility of MAP in risk stratification.

The prevalence of hypertension has been steadily increasing in Asia than in Western countries [23] However, only 54 % of the affected individuals are diagnosed, and only 42 % receive treatment [23] These findings underscore the urgent need to increase public awareness and improve hypertension management. Effective BP control is fundamental for preventing hypertension-related complications.

In this study, we demonstrated that MAP has a superior predictive ability for incident CVD than SBP, DBP, and PP. The MAP is often used in critical care settings to assess organ perfusion [24] Furthermore, recent research has shown that MAP is associated with a long-term risk of stroke [12] Although the mechanisms underlying the association between MAP and future cardiovascular risk remain uncertain, MAP reflects the average arterial pressure throughout the cardiac cycle [6] and may therefore capture sustained hemodynamic stress more accurately than SBP, DBP, or PP, which represent only specific phases of the cycle. Current hypertension guidelines primarily emphasize SBP and DBP, owing to their ease of measurement and robust evidence [19] However, our findings suggest that MAP has additional prognostic value and should be considered in routine cardiovascular risk assessments. These results support the routine assessment of MAP, along with SBP and DBP, in the evaluation of cardiovascular risk.

In our study, all BP indices were associated with the risk of CVD; however, PP demonstrated the lowest predictive ability compared with SBP, DBP, and MAP. This finding is consistent with those of previous studies that have also reported a relatively limited prognostic value of PP for future CVD risk compared with other BP metrics [16,17] The study by Mosley et al., [16] which included only participants who did not receive antihypertensive treatment, strongly supports our findings. Conversely, the study by Sesso et al., [15] which also excluded individuals on antihypertensive medications, stratified the analysis by age and reported that although PP had a limited predictive value compared to SBP and MAP for males <60 years, it exhibited superior predictive ability for CVD risk compared to MAP among those aged ≥60 years. The discrepancy between our findings and those of Sesso et al. [15] may be attributable to the younger age distribution of the participants in our study and that of Mosley et al. [16] When we limited our analysis to participants aged ≥50 years, the predictive power of MAP was less pronounced than that in the primary analysis, suggesting that the prognostic utility of BP indices may vary by age. Our findings and previous evidence can be interpreted within the physiological framework of the two hemodynamic components of blood pressure. In our relatively young cohort, the predominant pathophysiological mechanism of hypertension is likely an increase in peripheral vascular resistance, [25] best reflected by the stable hemodynamic component—MAP [6] In contrast, PP, representing the pulsatile component, increases substantially with advancing age owing to progressive arterial stiffening, [7] which may explain its limited prognostic relevance in this younger population.

We conducted a subgroup analyses stratifying individuals by gender and observed notable differences in the predictive utility of BP indices for CVD risk. A previous study established a stronger association between conventional risk factors, such as atherosclerotic plaque burden, and cardiovascular events in males [26] Conversely, although young females are generally protected against CVD, the risk in older females may exceed that observed in males [26,27] Estrogen plays a pivotal role in modulating vascular function and cardiovascular risk in females, [28] and menopause is a critical physiological transition. Although our study cohort was relatively young, it is likely that a proportion of middle-aged female participants had undergone menopause. Estrogen deficiency may increase peripheral vascular resistance and negatively affects cardiac output, [29,30] potentially altering the interpretation of MAP in postmenopausal females. These factors may have contributed to the attenuated predictive value of MAP in females in our analysis.

To contextualize the clinical implications of our findings, it should be noted that established cardiovascular risk models, such as the Framingham and JPHC scores, [31,32]. integrate multiple demographic and metabolic factors—including age, sex, and lipid profiles—to provide comprehensive risk estimation. Conversely, MAP reflects a single hemodynamic dimension of vascular load. Although MAP exhibited superior predictive ability compared with other BP indices in our analysis, its prognostic advantage may be attenuated among females and older individuals, in whom hormonal alterations and increased arterial stiffness may reduce vascular compliance and modify the relationship between BP and cardiovascular risk. Therefore, MAP may serve not as a substitute for multifactorial risk models, but rather as a simple and clinically complementary marker for cardiovascular risk stratification. The C-statistic of the full model in the present study was lower than that reported by the Framingham study. However, this discrepancy should be interpreted not as an indication of inferior model performance, but as a reflection of fundamental differences in the underlying population characteristics. First, our cohort was markedly younger than the Framingham cohort. Although age is a powerful predictor of CVD, the variance in risk attributable to age is limited in a younger and relatively homogeneous population, such as ours. This makes risk discrimination inherently more challenging. Second, the event rate in our study was low. The C-statistic is known to yield lower absolute values in low-event-rate populations, as it reflects the greater statistical difficulty of discriminating between cases and non-cases. As the C-statistic is highly dependent on the baseline risk profile of the population, direct comparisons of its absolute value across cohorts with different characteristics are inadvisable. Thus, the C-statistic obtained in our study reflects the model's discriminatory ability for the more challenging task of predicting future MACE in a young, low-risk population, a context distinct from that of the Framingham study.

This study has few limitations. First, the cohort was exclusively composed of Japanese individuals, which may have introduced selection bias and restricted the applicability of our results to other ethnic or geographic populations. The risk of CVD varies significantly by ethnicity [33] While Asians are known to have a higher risk of hypertension-related CVD than Western populations, [34] studies have also revealed differences in blood pressure profiles within Asia, specifically between East and South Asians [35] Given this heterogeneity, the optimal MAP threshold for predicting CVD risk found in our cohort may have limited applicability to other demographic groups. Therefore, future prospective studies in multi-ethnic populations are required to confirm the validity and assess the broader generalizability of our findings. Additionally, our study may be subject to the other selection bias, as a proportion of participants were excluded from the analysis due to unavailability of data or follow-up. To examine the potential impact of this exclusion, we compared baseline characteristics between participants included in the analysis and those excluded but who otherwise met the criterion of not receiving antihypertensive medication (Supplementary Table S12). Although statistically significant differences were observed for several variables, the absolute differences in key covariates, such as mean age, blood pressure, and body mass index, were small and unlikely to be clinically meaningful. Therefore, it is unlikely that this potential selection bias materially affected the main findings of our study. Second, as this was a retrospective observational study, causal relationships could not be established. Thirdly, because BP was measured at a single time point, measurement variability could not be completely excluded, which may have attenuated the observed associations. Finally, although information on medication use was available, we lacked specific data on pharmacological treatments, including glucose-, antihypertensive-, and lipid-lowering agents.

Nonetheless, this study has several strengths. To our knowledge, this is the first investigation to examine the long-term prognostic utility of various BP indices in relation to incident cardiovascular disease over a 13-year follow-up period in a substantial East Asian population cohort.

## 5 Conclusion

Our findings indicate that MAP outperformed SBP, DBP, and PP in predicting the 13-year incidence of CVD in the Japanese population. From a clinical perspective, a MAP threshold of 91.7 mmHg can identify individuals at high risk, suggesting that incorporating MAP into routine evaluation could enhance cardiovascular risk stratification. However, the prognostic advantage of MAP appeared attenuated in females and older adults, indicating that gender and age may modify its clinical utility. These results provide a strong rationale for incorporating MAP into clinical guidelines and public health screening to improve early risk classification and facilitate timely preventive interventions, particularly in younger populations.

**Figure:**
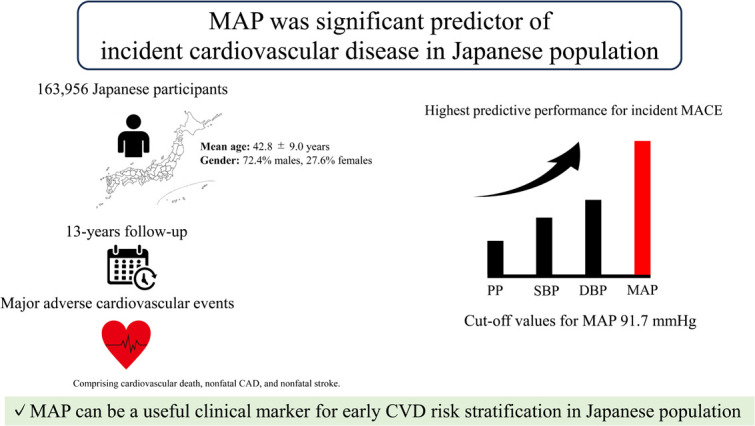
Central Illustlation: *Legend.*

## Funding

This research was supported by 10.13039/501100003478MHLW Comprehensive Research Project for Measures against Cardiovascular Diseases, Diabetes and Other Lifestyle Related Diseases Program, Grant Number JPMH 24FA1008. The sponsor was not involved in the collection, analysis and interpretation of data, and in the writing of the article.

## Statement of ethics

This cohort study was conducted according to the principles stated in Declaration of Helsinki and the protocol of the present study approved by the local ethics committee of the Panasonic Health Insurance Organization (approval number: 2021–001).

## CRediT authorship contribution statement

**Takahiro Ichikawa:** Writing – original draft, Visualization, Methodology, Investigation, Formal analysis. **Hiroshi Okada:** Writing – review & editing, Validation, Software, Project administration, Methodology, Investigation, Formal analysis, Data curation, Conceptualization. **Hanako Nakajima:** Writing – review & editing, Investigation. **Emi Ushigome:** Writing – review & editing, Investigation. **Masahide Hamaguchi:** Writing – review & editing, Investigation. **Kazushiro Kurogi:** Writing – review & editing, Resources, Investigation. **Hiroaki Murata:** Writing – review & editing, Resources, Investigation. **Eri Tsuda:** Writing – review & editing, Resources, Investigation. **Naoki Yoshida:** Writing – review & editing, Resources, Investigation. **Masato Ito:** Writing – review & editing, Resources, Investigation. **Michiaki Fukui:** Writing – review & editing, Supervision, Investigation.

## Declaration of competing interest

We wish to draw the attention of the Editor to the following facts which may be considered as potential conflicts of interest and to significant financial contributions to this work.

Hiroshi Okada received grants from Japan Diabetes Foundation. Emi Ushigome received grants from The Food and Health Research Chair to which Emi Ushigome belongs is a joint research chair funded by Yoshinoya Holdings Co., Ltd. and Taiyo Kagaku Co., Ltd. Masahide Hamaguchi received grants from AstraZeneca K.K., Ono Pharma Co. Ltd., and Kowa Pharma Co. Ltd. Michiaki Fukui received grants from Ono Pharma Co. Ltd., Oishi Kenko inc., Yamada Bee Farm, Nippon Boehringer Ingelheim Co. Ltd., Kissei Pharma Co. Ltd., Mitsubishi Tanabe Pharma Corp., Daiichi Sankyo Co. Ltd., Sanofi K.K., Takeda Pharma Co. Ltd., Astellas Pharma Inc., MSD K.K., Kyowa Kirin Co., Ltd., Sumitomo Dainippon Pharma Co., Ltd., Kowa Pharma Co. Ltd., Novo Nordisk Pharma Ltd., Sanwa Kagaku Kenkyusho CO., Ltd., Eli Lilly, Japan, K.K., Taisho Pharma Co., Ltd., Terumo Corp., Tejin Pharma Ltd., Nippon Chemiphar Co., Ltd., Abbott Japan Co. Ltd., Johnson & Johnson K.K. Medical Co., and TERUMO CORPORATION. The other authors declare that they have no competing interests.

## Appendix Supplementary materials

Image, application 1

Image, application 2
