# Supplementary material for: Blood pressure indices for predicting incident cardiovascular disease: A 13-year follow-up study in Japanese population
Source: Am J Prev Cardiol. 2025 Nov 3;24:101341. doi: 10.1016/j.ajpc.2025.101341 (PMC12746281; doi:10.1016/j.ajpc.2025.101341)

Supplementary Figure 1.

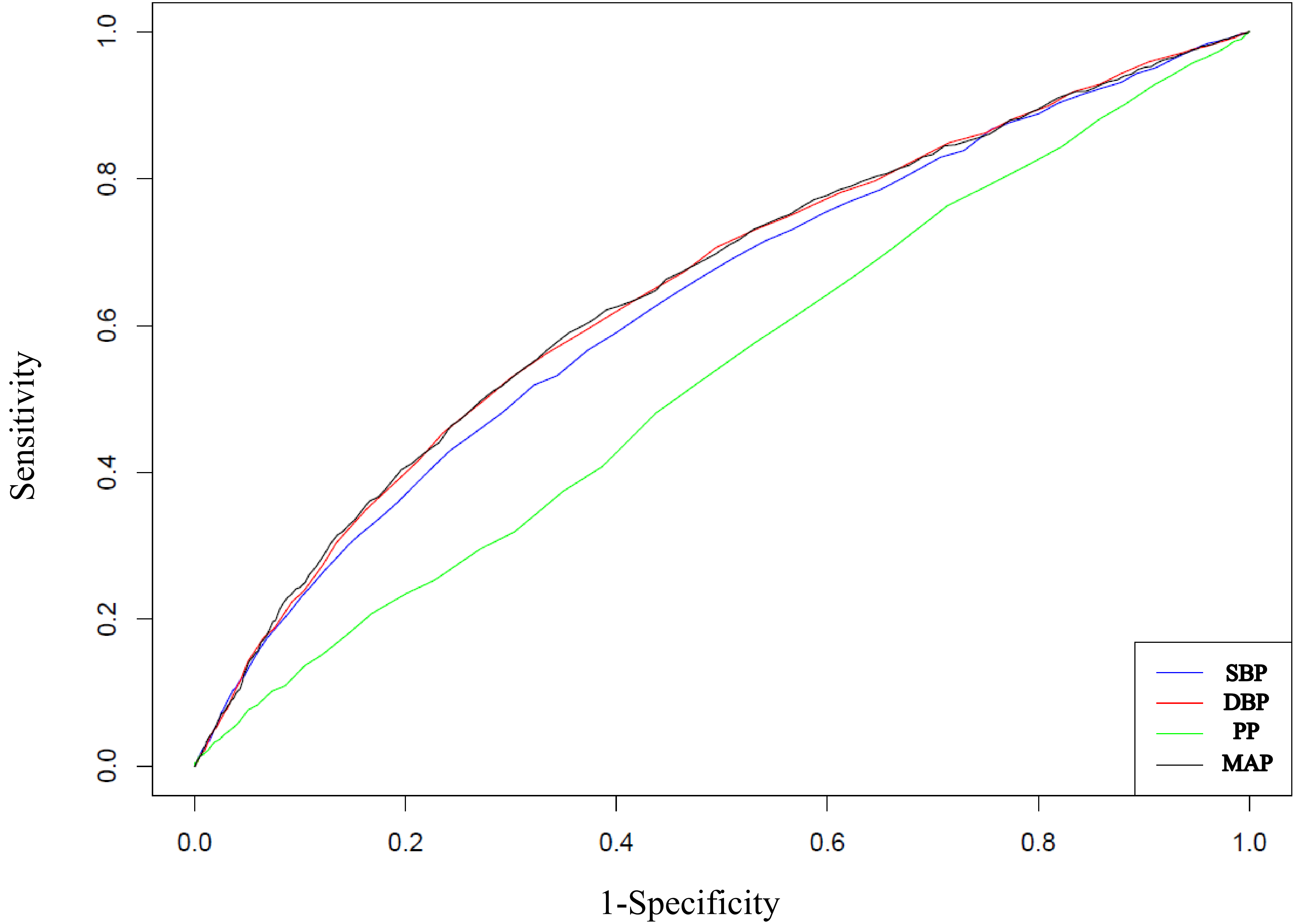

Supplementary Figure 2.

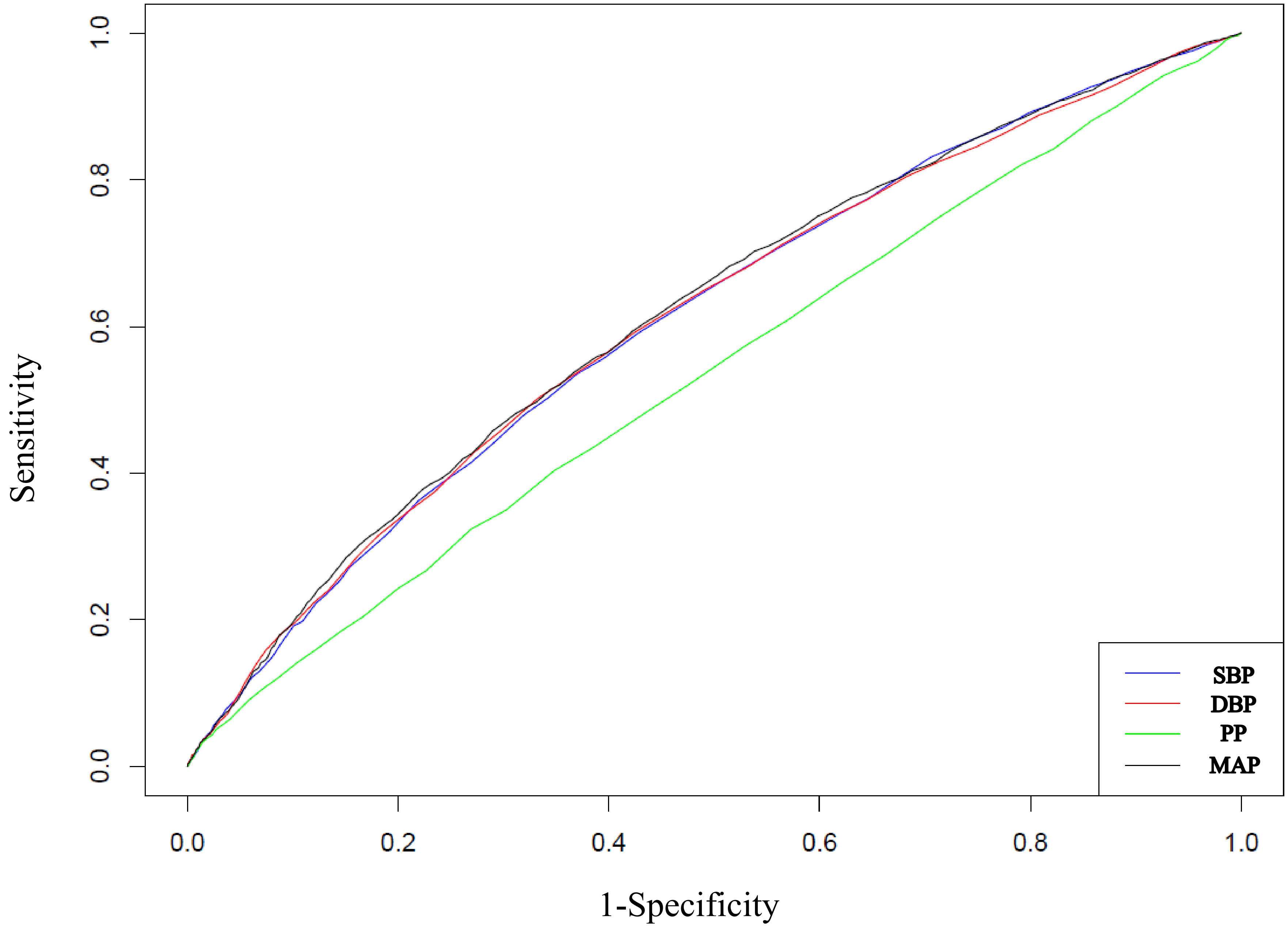

Supplementary Figure 3.

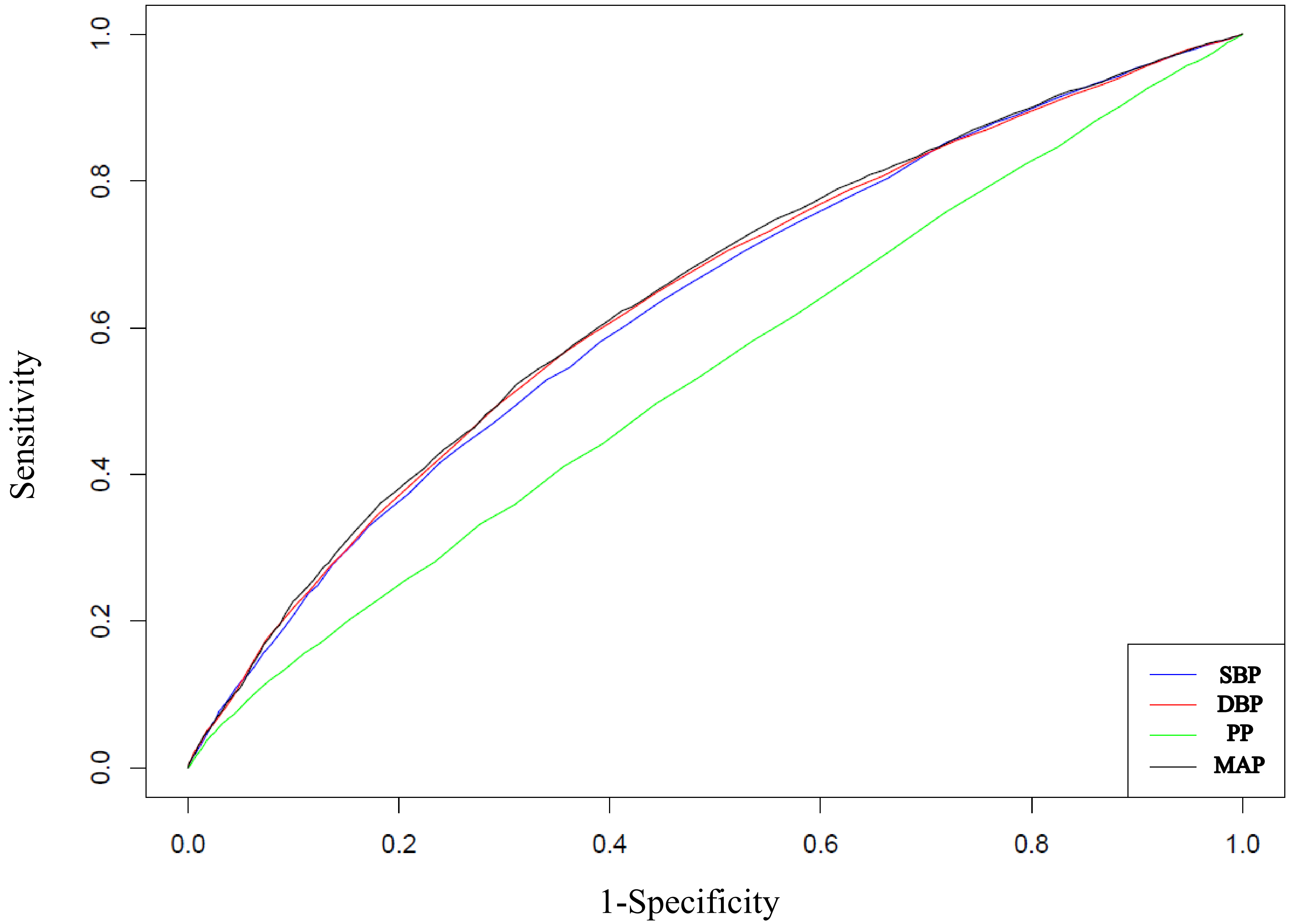

Supplementary Figure 4.

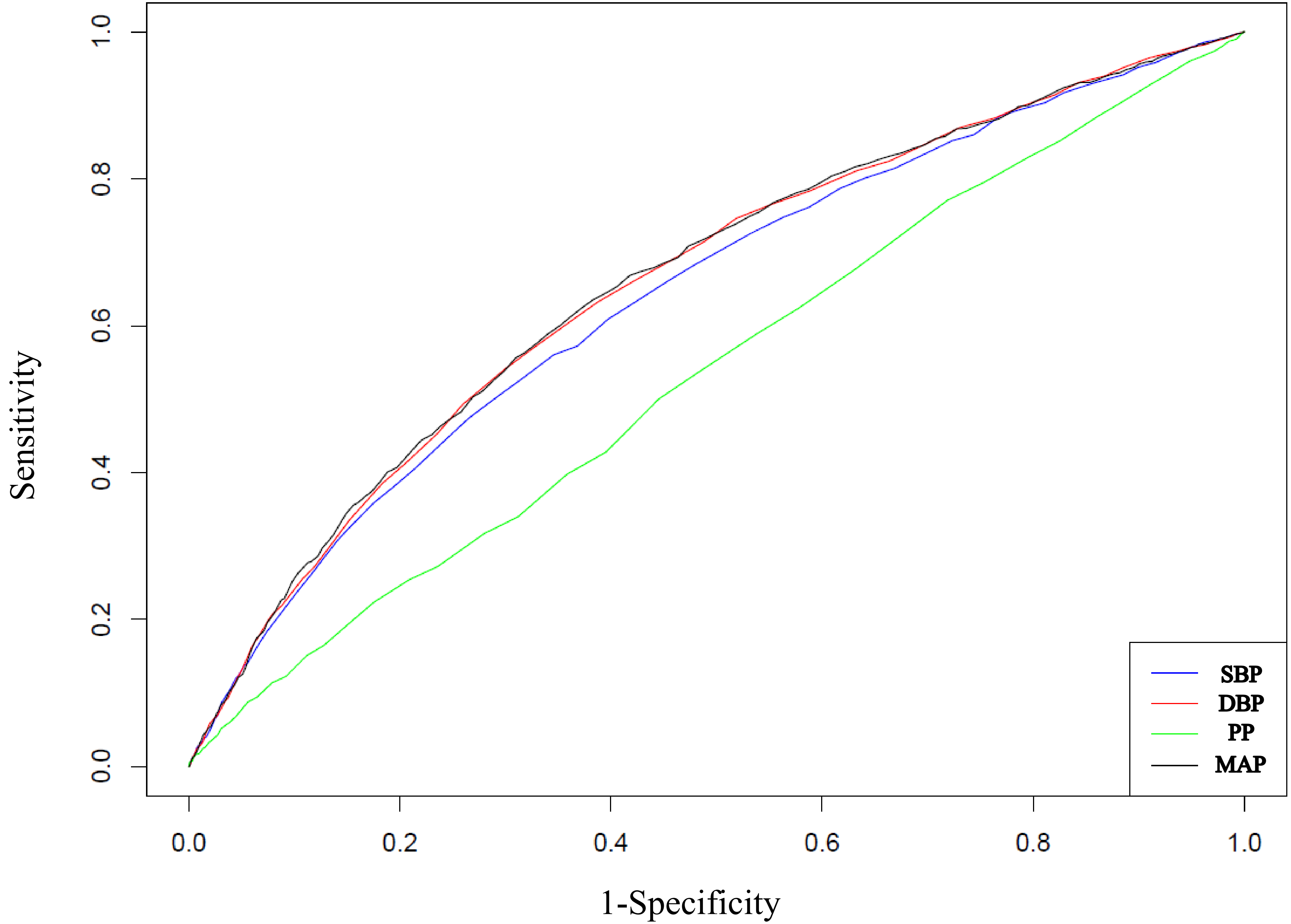

Supplementary Figure 5.

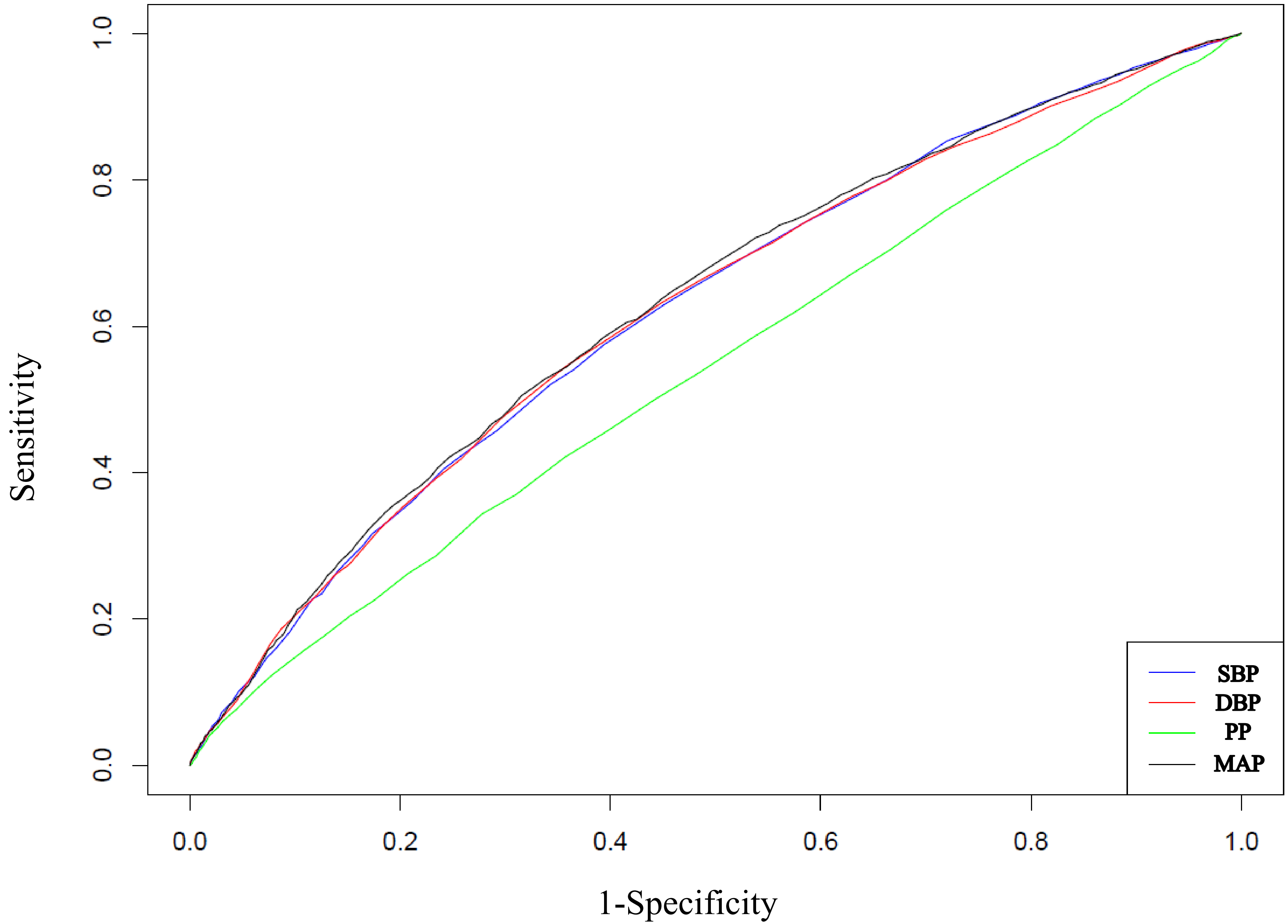

Supplement: Supplementary file 1 [file mmc1.pdf]
