# Supplementary material for: Blood pressure indices for predicting incident cardiovascular disease: A 13-year follow-up study in Japanese population
Source: Am J Prev Cardiol. 2025 Nov 3;24:101341. doi: 10.1016/j.ajpc.2025.101341 (PMC12746281; doi:10.1016/j.ajpc.2025.101341)
Supplement: Supplementary file 2 [file mmc2.docx]

Supplementary Table 1. Baseline characteristics of the overall study population

|  | ALL | Anti-hypertensive drugs (-) | Anti-hypertensive drugs (+) |
| --- | --- | --- | --- |
| N | 175,370 | 163,956 | 11,414 |
| Age (y) | 43.3 (9.0) | 42.8 (9.0) | 51.1 (6.2) |
| Gender (males/females) (%) | 128,890 /46,480 (73.5/26.5) | 118,753/45,203 (72.4/27.6) | 10,137/1277 (88.8/11.2) |
| Body mass index (kg/m^2^) | 23.0 (3.6) | 22.8 (3.4) | 25.7 (4.2) |
| Systolic blood pressure (mmHg) | 118.6 (14.8) | 117.7 (14.4) | 131.6 (14.9) |
| Diastolic blood pressure (mmHg) | 73.8 (11.2) | 73.1 (10.9) | 83.8 (10.1) |
| Pulse pressure (mmHg) | 44.8 (8.9) | 44.6 (8.8) | 47.8 (10.3) |
| Mean arterial pressure (mmHg) | 88.8 (11.8) | 88.0 (11.5) | 99.7 (10.9) |
| Triglycerides (mg/dL) | 109.9 (90.8) | 107.3 (88.5) | 146.3 (112.8) |
| HDL cholesterol (mg/dL) | 60.6 (15.3) | 60.9 (15.3) | 56.5 (14.8) |
| LDL cholesterol (mg/dL) | 122.2 (31.6) | 122.0 (31.7) | 124.7 (29.7) |
| Fasting plasma glucose (mg/dl) | 94.3 (17.3) | 93.5 (16.3) | 105.5 (25.0) |
| Glucose-lowering medications (+/-) | 3,579/171,791 (2.0/98.0) | 2,306/161,650 (1.4/98.6) | 1,273/10,141 (11.2/88.8) |
| Lipid-lowering medications (+/-) | 5,620/169,750 (3.2/96.8) | 3,437/160,519 (2.1/97.9) | 2,183/9,231 (19.1/80.9) |
| Smoking (none/past/current)  (%) | 96,895/23,281/55,194  (55.3/13.3/31.5) | 91,224/21,033/51,699  (55.6/12.8/31.5) | 5,671/2,248/3,495  (49.7/19.7/30.6) |
| Drinker (+/-) (%) | 40,369/135,001 (23.0/77.0) | 36,030/127,926 (22.0/78.0) | 4,339/7,075 (23.0/77.0) |
| Physical exercise (+/-) (%) | 30,464/144,906 (17.4/82.6) | 28,084/135,872 (17.1/82.9) | 2,380/9,034 (20.9/79.2) |

Data are presented as mean (standard deviation, or percentage) or absolute number.

Abbreviations: LDL, low-density lipoprotein; HDL, high-density lipoprotein.

Supplementary Table 2. Examination of the model with incident MACE in participants not receiving antihypertensive medications.

(8 variables to be forced in + additional blood pressure indices)

| Variables added | -2LL_0 | -2LL_1 | AIC | -2(LL_0-LL_1) | df | p value |
| --- | --- | --- | --- | --- | --- | --- |
| SBP | 82,123.77 | 81,904.99 | 81,924.99 | 218.78 | 1 | <0.001 |
| DBP |  | 81,885.77 | 81,905.77 | 238.01 | 1 | <0.001 |
| PP |  | 82,093.15 | 82,113.15 | 30.63 | 1 | <0.001 |
| MAP |  | 81,865.37 | 81,885.37 | 258.40 | 1 | <0.001 |

8 variables; gender, age, body mass index, drinking habits, smoking status, physical exercise, the presence of IFG, and the presence of dyslipidemia at baseline.

-2LL_0; The -2 log likelihood of the model with the 8 variables to be forced in as main effects

-2LL_1; The -2 log likelihood of the model with the above 8 variables + additional blood pressure indices

-2(LL_0-LL_1); Test statistic

df; Degrees of freedom of the test statistic (difference in the number of parameters)

Abbreviation: MACE, major adverse cardiovascular event; SBP, systolic blood pressure; DBP, diastolic blood pressure; PP, pulse pressure; MAP, mean arterial pressure.

Supplementary Table 3. Examination of the model with incident stroke in participants not receiving antihypertensive medications.

(8 variables to be forced in + additional blood pressure indices)

| Variables added | -2LL_0 | -2LL_1 | AIC | -2(LL_0-LL_1) | df | p value |
| --- | --- | --- | --- | --- | --- | --- |
| SBP | 28,048.71 | 27,927.8 | 27,947.8 | 120.91 | 1 | <0.001 |
| DBP |  | 27,898.2 | 27,918.2 | 150.51 | 1 | <0.001 |
| PP |  | 28,038.41 | 28,058.41 | 10.30 | 1 | 0.001 |
| MAP |  | 27,894.1 | 27,914.1 | 154.62 | 1 | <0.001 |

8 variables; gender, age, body mass index, drinking habits, smoking status, physical exercise, the presence of IFG, and the presence of dyslipidemia at baseline.

-2LL_0; The -2 log likelihood of the model with the 8 variables to be forced in as main effects

-2LL_1; The -2 log likelihood of the model with the above 8 variables + additional blood pressure indices

-2(LL_0-LL_1); Test statistic

df; Degrees of freedom of the test statistic (difference in the number of parameters)

Abbreviation: SBP, systolic blood pressure; DBP, diastolic blood pressure; PP, pulse pressure; MAP, mean arterial pressure.

Supplementary Table 4. Examination of the model with incident CAD in participants not receiving antihypertensive medications.

(8 variables to be forced in + additional blood pressure indices)

| Variables added | -2LL_0 | -2LL_1 | AIC | -2(LL_0-LL_1) | df | p value |
| --- | --- | --- | --- | --- | --- | --- |
| SBP | 54,739.19 | 54,643.13 | 54,663.13 | 96.05 | 1 | <0.001 |
| DBP |  | 54,652.4 | 54,672.4 | 86.79 | 1 | <0.001 |
| PP |  | 54,717.33 | 54,737.33 | 21.86 | 1 | 0.001 |
| MAP |  | 54,637.34 | 54,657.34 | 101.85 | 1 | <0.001 |

8 variables; gender, age, body mass index, drinking habits, smoking status, physical exercise, the presence of IFG, and the presence of dyslipidemia at baseline.

-2LL_0; The -2 log likelihood of the model with the 8 variables to be forced in as main effects

-2LL_1; The -2 log likelihood of the model with the above 8 variables + additional blood pressure indices

-2(LL_0-LL_1); Test statistic

df; Degrees of freedom of the test statistic (difference in the number of parameters)

Abbreviation: CAD, coronary artery disease; SBP, systolic blood pressure; DBP, diastolic blood pressure; PP, pulse pressure; MAP, mean arterial pressure.

Supplementary Table 5. Unadjusted hazard ratios and multivariate adjusted hazard ratios for MACE, stroke, CAD in participants not receiving antihypertensive medications, stratified by gender. (per SD)

| Males | | | | | | |
| --- | --- | --- | --- | --- | --- | --- |
| MACE | Crude | | Model 1 | | Model 2 | |
|  | Hazard ratios (95% CI)  C-Statistics (95% CI) | *p* | Hazard ratios (95% CI)  C-Statistics (95% CI) | *p* | Hazard ratios (95% CI)  C-Statistics (95% CI) | *p* |
| SBP | 1.45 (1.41-1.50)  0.643 (0.626-0.660) | <0.001 | 1.40 (1.35-1.45)  0.643 (0.626-0.660) | <0.001 | 1.35 (1.30-1.40)  0.649 (0.632-0.666) | <0.001 |
| DBP | 1.51 (1.46-1.56)  0.645 (0.628-0.662) | <0.001 | 1.47 (1.42-1.52)  0.645 (0.628-0.662) | <0.001 | 1.42 (1.37-1.47)  0.651 (0.634-0.669) | <0.001 |
| PP | 1.11 (1.07-1.15)  0.627 (0.611-0.644) | <0.001 | 1.07 (1.03-1.11)  0.627 (0.611-0.644) | <0.001 | 1.06 (1.02-1.10)  0.636 (0.619-0.653) | 0.001 |
| MAP | 1.52 (1.47-1.57)  0.647 (0.630-0.664) | <0.001 | 1.47 (1.42-1.53)  0.647 (0.630-0.664) | <0.001 | 1.42 (1.37-1.47)  0.652 (0.635-0.669) | <0.001 |
| Stroke | Crude | | Model 1 | | Model 2 | |
|  | Hazard ratios (95% CI)  C-Statistics (95% CI) | *p* | Hazard ratios (95% CI)  C-Statistics (95% CI) | *p* | Hazard ratios (95% CI)  C-Statistics (95% CI) | *p* |
| SBP | 1.52 (1.44-1.61)  0.609 (0.575-0.644) | <0.001 | 1.48 (1.39-1.56)  0.636 (0.606-0.666) | <0.001 | 1.44 (1.36-1.53)  0.638 (0.608-0.668) | <0.001 |
| DBP | 1.62 (1.53-1.71)  0.634 (0.600-0.667) | <0.001 | 1.59 (1.50-1.68)  0.645 (0.616-0.675) | <0.001 | 1.55 (1.46-1.65)  0.647 (0.617-0.677) | <0.001 |
| PP | 1.11 (1.04-1.17)  0.515 (0.481-0.549) | 0.001 | 1.07 (1.01-1.14)  0.608 (0.579-0.637) | 0.028 | 1.06 (1.00-1.13)  0.612 (0.583-0.641) | 0.046 |
| MAP | 1.61 (1.52-1.70)  0.632 (0.598-0.666) | <0.001 | 1.58 (1.49-1.67)  0.645 (0.615-0.675) | <0.001 | 1.54 (1.45-1.64)  0.647 (0.617-0.677) | <0.001 |
| CAD | Crude | | Model 1 | | Model 2 | |
|  | Hazard ratios (95% CI)  C-Statistics (95% CI) | *p* | Hazard ratios (95% CI)  C-Statistics (95% CI) | *p* | Hazard ratios (95% CI)  C-Statistics (95% CI) | *p* |
| SBP | 1.41 (1.35-1.46)  0.587 (0.563-0.611) | <0.001 | 1.34 (1.29-1.40)  0.643 (0.623-0.664) | <0.001 | 1.29 (1.23-1.35)  0.652 (0.632-0.673) | <0.001 |
| DBP | 1.44 (1.38-1.50)  0.596 (0.573-0.620) | <0.001 | 1.38 (1.32-1.45)  0.643 (0.622-0.663) | <0.001 | 1.33 (1.27-1.39)  0.652 (0.632-0.672) | <0.001 |
| PP | 1.12 (1.08-1.17)  0.518 (0.494-0.543) | <0.001 | 1.08 (1.03-1.12)  0.634 (0.613-0.654) | 0.001 | 1.07 (1.02-1.11)  0.645 (0.625-0.665) | 0.003 |
| MAP | 1.45 (1.39-1.51)  0.599 (0.575-0.622) | <0.001 | 1.39 (1.34-1.46)  0.644 (0.624-0.665) | <0.001 | 1.34 (1.28-1.40)  0.653 (0.633-0.674) | <0.001 |
| Females | | | | | | |
| MACE | Crude | | Model 1 | | Model 2 | |
|  | Hazard ratios (95% CI)  C-Statistics (95% CI) | *p* | Hazard ratios (95% CI)  C-Statistics (95% CI) | *p* | Hazard ratios (95% CI)  C-Statistics (95% CI) | *p* |
| SBP | 1.38 (1.25-1.51)  0.641 (0.585-0.696) | <0.0001 | 1.40 (1.26-1.55)  0.641 (0.585-0.696) | <0.001 | 1.36 (1.23-1.51)  0.650 (0.594-0.705) | <0.001 |
| DBP | 1.37 (1.24-1.52)  0.638 (0.581-0.694) | <0.001 | 1.37 (1.22-1.53)  0.638 (0.581-0.694) | <0.0001 | 1.33 (1.19-1.49)  0.646 (0.589-0.703) | <0.001 |
| PP | 1.21 (1.09-1.34)  0.627 (0.572-0.683) | <0.001 | 1.20 (1.08-1.33)  0.627 (0.572-0.683) | 0.001 | 1.18 (1.06-1.31)  0.637 (0.581-0.693) | 0.002 |
| MAP | 1.39 (1.26-1.54)  0.641 (0.585-0.697) | <0.001 | 1.40 (1.26-1.56)  0.641 (0.585-0.697) | <0.001 | 1.37 (1.23-1.53)  0.649 (0.593-0.706) | <0.001 |
| Stroke | Crude | | Model 1 | | Model 2 | |
|  | Hazard ratios (95% CI)  C-Statistics (95% CI) | *p* | Hazard ratios (95% CI)  C-Statistics (95% CI) | *p* | Hazard ratios (95% CI)  C-Statistics (95% CI) | *p* |
| SBP | 1.46 (1.27-1.69)  0.607 (0.509-0.705) | <0.001 | 1.45 (1.24-1.71)  0.661 (0.576-0.747) | <0.001 | 1.41 (1.20-1.67)  0.672 (0.587-0.757) | <0.001 |
| DBP | 1.51 (1.29-1.77)  0.611 (0.513-0.709) | <0.001 | 1.48 (1.25-1.76)  0.664 (0.578-0.749) | <0.001 | 1.44 (1.21-1.72)  0.675 (0.590-0.760) | <0.001 |
| PP | 1.21 (1.02-1.42)  0.543 (0.446-0.641) | 0.026 | 1.17 (0.99-1.39)  0.648 (0.563-0.734) | 0.065 | 1.15 (0.98-1.37)  0.659 (0.573-0.745) | 0.094 |
| MAP | 1.51 (1.30-1.75)  0.614 (0.515-0.713) | <0.001 | 1.50 (1.27-1.77)  0.665 (0.580-0.751) | <0.001 | 1.46 (1.23-1.73)  0.676 (0.591-0.761) | <0.001 |
| CAD | Crude | | Model 1 | | Model 2 | |
|  | Hazard ratios (95% CI)  C-Statistics (95% CI) | *p* | Hazard ratios (95% CI)  C-Statistics (95% CI) | *p* | Hazard ratios (95% CI)  C-Statistics (95% CI) | *p* |
| SBP | 1.34 (1.19-1.51)  0.599 (0.527-0.671) | <0.001 | 1.35 (1.18-1.54)  0.635 (0.564-0.706) | <0.001 | 1.32 (1.16-1.51)  0.639 (0.567-0.711) | <0.001 |
| DBP | 1.30 (1.14-1.48)  0.580 (0.506-0.655) | <0.001 | 1.28 (1.11-1.48)  0.629 (0.556-0.702) | 0.001 | 1.25 (1.09-1.45)  0.632 (0.558-0.706) | 0.002 |
| PP | 1.22 (1.07-1.38)  0.548 (0.472-0.624) | 0.002 | 1.21 (1.06-1.38)  0.626 (0.554-0.698) | 0.004 | 1.20 (1.05-1.37)  0.631 (0.559-0.703) | 0.006 |
| MAP | 1.34 (1.17-1.51)  0.593 (0.520-0.667) | <0.001 | 1.33 (1.16-1.53)  0.632 (0.561-0.705) | <0.001 | 1.30 (1.14-1.50)  0.636 (0.563-0.709) | <0.001 |

Data are presented as hazard ratios (95% confidence intervals).

Model 1 was adjusted for BMI, drinking habits, smoking status, and physical exercise.

Model 2 was adjusted for BMI, drinking habits, smoking status, physical exercise, the presence of IFG, and the presence of dyslipidemia.

Abbreviations: SD, standard deviation; CI, confidence intervals; SBP, systolic blood pressure; DBP, diastolic blood pressure; PP, pulse pressure; MAP, mean arterial pressure; MACE, major adverse cardiovascular events; CAD, coronary artery disease; BMI, Body mass index; IFG, impaired fasting glucose.

Supplementary Table 6. Comparison of area under the curve of SBP, DBP, PP, and MAP with incident MACE, stroke, and CAD in participants not receiving antihypertensive medications, stratified by gender.

| **Males** | | | | | | |
| --- | --- | --- | --- | --- | --- | --- |
| **MACE** | vs. SBP | | vs. DBP | | vs. PP | |
|  | difference  value | 95% CI  P value | difference  value | 95% CI  P value | difference  value | 95% CI  P value |
| SBP | reference | | - | | - | |
| DBP | 0.009 | 0.001 to 0.017  P = 0.04 | reference | | - | |
| PP | -0.083 | -0.093 to -0.073  P < 0.001 | -0.091 | -0.108 to -0.075  P < 0.001 | reference | |
| MAP | 0.013 | 0.007 to 0.018  P < 0.001 | 0.004 | 0.0004 to 0.007  P = 0.028 | 0.095 | 0.081 to 0.109  P < 0.001 |
| **Stroke** | vs. SBP | | vs. DBP | | vs. PP | |
|  | difference  value | 95% CI  P value | difference  value | 95% CI  P value | difference  value | 95% CI  P value |
| SBP | reference | | - | | - | |
| DBP | 0.021 | 0.008 to 0.034  P = 0.002 | reference | | - | |
| PP | -0.103 | -0.119 to -0.088  P < 0.001 | -0.123 | -0.150 to -0.101  P < 0.001 | reference | |
| MAP | 0.022 | 0.014 to 0.031  P < 0.001 | 0.001 | -0.004 to 0.006  P = 0.648 | 0.125 | 0.105 to 0.147  P < 0.001 |
| **CAD** | vs. SBP | | vs. DBP | | vs. PP | |
|  | difference  value | 95% CI  P value | difference  value | 95% CI  P value | difference  value | 95% CI  P value |
| SBP | reference | | - | | - | |
| DBP | -0.0002 | -0.010 to 0.009  P = 0.968 | reference | | - | |
| PP | -0.070 | -0.082 to -0.058  P < 0.001 | -0.070 | -0.089 to -0.049  P < 0.001 | reference | |
| MAP | 0.006 | -0.0001 to 0.012  P = 0.058 | 0.006 | 0.003 to 0.010  P < 0.001 | 0.076 | 0.058 to 0.093  P < 0.001 |
| **Females** | | | | | | |
| **MACE** | vs. SBP | | vs. DBP | | vs. PP | |
|  | difference  value | 95% CI  P value | difference  value | 95% CI  P value | difference  value | 95% CI  P value |
| SBP | reference | | - | | - | |
| DBP | -0.003 | -0.024 to 0.017  P = 0.754 | reference | | - | |
| PP | -0.067 | -0.096 to -0.037  P < 0.001 | -0.063 | -0.110 to -0.019  P = 0.004 | reference | |
| MAP | 0.003 | -0.002 to 0.015  P = 0.622 | 0.007 | -0.002 to 0.015  P = 0.122 | 0.070 | 0.032 to 0.110  P < 0.001 |
| **Stroke** | vs. SBP | | vs. DBP | | vs. PP | |
|  | difference  value | 95% CI  P value | difference  value | 95% CI  P value | difference  value | 95% CI  P value |
| SBP | reference | | - | | - | |
| DBP | -0.005 | -0.042 to 0.026  P = 0.728 | reference | | - | |
| PP | -0.063 | -0.113 to -0.012  P = 0.016 | -0.057 | -0.132 to 0.020  P = 0.154 | reference | |
| MAP | 0.002 | -0.020 to 0.021  P = 0.894 | 0.007 | -0.006 to 0.022  P = 0.276 | 0.064 | -0.004 to 0.129  P = 0.066 |
| **CAD** | vs. SBP | | vs. DBP | | vs. PP | |
|  | difference  value | 95% CI  P value | difference  value | 95% CI  P value | difference  value | 95% CI  P value |
| SBP | reference | | - | | - | |
| DBP | -0.003 | -0.030 to 0.026  P = 0.836 | reference | | - | |
| PP | -0.074 | -0.110 to -0.036  P < 0.001 | -0.071 | -0.129 to -0.014  P = 0.02 | reference | |
| MAP | 0.004 | -0.013 to 0.022  P = 0.582 | 0.007 | -0.004 to 0.018  P = 0.194 | 0.078 | 0.028 to 0.129  P = 0.008 |

Abbreviations: MACE, major adverse cardiovascular event; CAD, coronary arterial disease; SBP, systolic blood pressure; DBP, diastolic blood pressure; PP, pulse pressure; MAP, mean arterial pressure

Supplementary Table 7. Unadjusted hazard ratios and multivariate adjusted hazard ratios for MACE, stroke, CAD in all participants, regardless of their antihypertensive medication status. (per SD)

| MACE | Crude | | Model 1 | | Model 2 | |
| --- | --- | --- | --- | --- | --- | --- |
|  | Hazard ratios (95% CI)  C-Statistics (95% CI) | *p* | Hazard ratios (95% CI)  C-Statistics (95% CI) | *p* | Hazard ratios (95% CI)  C-Statistics (95% CI) | *p* |
| SBP | 1.52 (1.49-1.56)  0.632 (0.616-0.648) | <0.001 | 1.29 (1.25-1.33)  0.692 (0.678-0.705) | <0.001 | 1.26 (1.23-1.30)  0.696 (0.682-0.710) | <0.001 |
| DBP | 1.60 (1.55-1.64)  0.645 (0.629-0.661) | <0.001 | 1.33 (1.29-1.37)  0.693 (0.680-0.707) | <0.001 | 1.31 (1.27-1.35)  0.698 (0.684-0.711) | <0.001 |
| PP | 1.16 (1.13-1.20)  0.534 (0.517-0.552) | <0.001 | 1.09 (1.06-1.13)  0.680 (0.667-0.694) | <0.001 | 1.08 (1.05-1.11)  0.686 (0.673-0.700) | <0.001 |
| MAP | 1.59 (1.55-1.63)  0.648 (0.632-0.664) | <0.001 | 1.34 (1.30-1.38)  0.694 (0.681-0.708) | <0.001 | 1.31 (1.27-1.35)  0.698 (0.685-0.712) | <0.001 |
| Stroke | Crude | | Model 1 | | Model 2 | |
|  | Hazard ratios (95% CI)  C-Statistics (95% CI) | *p* | Hazard ratios (95% CI)  C-Statistics (95% CI) | *p* | Hazard ratios (95% CI)  C-Statistics (95% CI) | *p* |
| SBP | 1.58 (1.51-1.65)  0.643 (0.615-0.671) | <0.001 | 1.37 (1.31-1.44)  0.683 (0.659-0.707) | <0.0001 | 1.36 (1.30-1.42)  0.684 (0.660-0.708) | <0.001 |
| DBP | 1.69 (1.61-1.76)  0.664 (0.637-0.692) | <0.001 | 1.46 (1.39-1.54)  0.689 (0.666-0.713) | <0.0001 | 1.45 (1.37-1.52)  0.690 (0.667-0.714) | <0.001 |
| PP | 1.16 (1.11-1.22)  0.533 (0.504-0.562) | <0.001 | 1.10 (1.04-1.15)  0.663 (0.640-0.686) | 0.0002 | 1.09 (1.04-1.14)  0.665 (0.642-0.689) | 0.001 |
| MAP | 1.67 (1.60-1.74)  0.664 (0.637-0.692) | <0.001 | 1.45 (1.38-1.52)  0.689 (0.666-0.713) | <0.0001 | 1.44 (1.37-1.51)  0.690 (0.667-0.714) | <0.001 |
| CAD | Crude | | Model 1 | | Model 2 | |
|  | Hazard ratios (95% CI)  C-Statistics (95% CI) | *p* | Hazard ratios (95% CI)  C-Statistics (95% CI) | *p* | Hazard ratios (95% CI)  C-Statistics (95% CI) | *p* |
| SBP | 1.48 (1.44-1.53)  0.625 (0.605-0.644) | <0.001 | 1.24 (1.19-1.28)  0.692 (0.676-0.709) | <0.001 | 1.21 (1.17-1.25)  0.698 (0.682-0.715) | <0.001 |
| DBP | 1.53 (1.48-1.58)  0.632 (0.612-0.651) | <0.001 | 1.25 (1.20-1.29)  0.692 (0.675-0.708) | <0.001 | 1.22 (1.17-1.27)  0.698 (0.681-0.714) | <0.001 |
| PP | 1.18 (1.14-1.22)  0.537 (0.515-0.558) | <0.001 | 1.10 (1.06-1.14)  0.685 (0.669-0.701) | <0.001 | 1.08 (1.05-1.12)  0.692 (0.676-0.709) | <0.001 |
| MAP | 1.53 (1.49-1.58)  0.636 (0.617-0.656) | <0.001 | 1.26 (1.21-1.31)  0.693 (0.676-0.709) | <0.001 | 1.23 (1.19-1.28)  0.699 (0.682-0.715) | <0.001 |

Data are presented as hazard ratios (95% confidence intervals).

Model 1 was adjusted for gender, age, BMI, drinking habits, smoking status, and physical exercise.

Model 2 was adjusted for gender, age, BMI, drinking habits, smoking status, physical exercise, the presence of IFG, and the presence of dyslipidemia.

Abbreviations: SD, standard deviation; CI, confidence intervals; SBP, systolic blood pressure; DBP, diastolic blood pressure; PP, pulse pressure; MAP, mean arterial pressure; MACE, major adverse cardiovascular events; CAD, coronary artery disease; BMI, Body mass index; IFG, impaired fasting glucose.

Supplementary Table 8. The area under the curve and optimal cut-off values with incident MACE, stroke, and CAD in all participants, regardless of their antihypertensive medication status.

| **MACE** | | | | | | | | |
| --- | --- | --- | --- | --- | --- | --- | --- | --- |
|  | AUC (95% CI) | Cut-off value | Sensitivity | Specificity | PPV | NPV | PLR | NLR |
| SBP | 0.629 (0.621-0.640) | 121 | 58.0 % | 60.9 % | 7.6 % | 96.3 % | 1.485 | 0.689 |
| DBP | 0.635 (0.626-0.646) | 77 | 56.3 % | 64.6 % | 8.1 % | 96.4 % | 1.590 | 0.676 |
| PP | 0.538 (0.530-0.548) | 49 | 33.2 % | 72.3 % | 6.2 % | 95.1 % | 1.199 | 0.924 |
| MAP | 0.640 (0.631-0.651) | 92.7 | 54.5 % | 66.7 % | 8.3 % | 96.4 % | 1.636 | 0.683 |
| **Stroke** | | | | | | | | |
|  | AUC (95% CI) | Cut-off value | Sensitivity | Specificity | PPV | NPV | PLR | NLR |
| SBP | 0.643 (0.629-0.657) | 123 | 56.0 % | 65.4 % | 2.8 % | 98.8 % | 1.621 | 0.672 |
| DBP | 0.659 (0.644-0.671) | 76 | 63.2 % | 61.3 % | 2.8 % | 98.9 % | 1.632 | 0.601 |
| PP | 0.537 (0.519-0.552) | 45 | 50.0 % | 55.5 % | 2.0 % | 98.4 % | 1.123 | 0.901 |
| MAP | 0.662 (0.645-0.673) | 91.3 | 63.5 % | 61.7 % | 2.9 % | 99.0 % | 1.658 | 0.591 |
| **CAD** | | | | | | | | |
|  | AUC (95% CI) | Cut-off value | Sensitivity | Specificity | PPV | NPV | PLR | NLR |
| SBP | 0.622 (0.611-0.631) | 121 | 57.5 % | 60.6 % | 5.4 % | 97.3 % | 1.459 | 0.702 |
| DBP | 0.621 (0.612-0.630) | 77 | 54.6 % | 64.2 % | 5.7 % | 97.3 % | 1.524 | 0.708 |
| PP | 0.542 (0.532-0.554) | 49 | 34.3 % | 72.2 % | 4.6 % | 96.5 % | 1.237 | 0.909 |
| MAP | 0.628 (0.619-0.637) | 91.0 | 58.4 % | 60.7 % | 5.5 % | 97.4 % | 1.487 | 0.685 |

Abbreviations: MACE, major adverse cardiovascular event; CAD, coronary artery disease; AUC, area under the curve; PPV, positive predictive value; NPV, negative predictive value; PLR, positive likelihood ratio; NLR, negative likelihood ratio.

Supplementary Table 9. Comparison of area under the curve of SBP, DBP, PP, and MAP with incident MACE, stroke, and CAD in all participants, regardless of their antihypertensive medication status.

| **MACE** | vs. SBP | | vs. DBP | | vs. PP | |
| --- | --- | --- | --- | --- | --- | --- |
|  | difference  value | 95% CI  P value | difference  value | 95% CI  P value | difference  value | 95% CI  P value |
| SBP | reference | | - | | - | |
| DBP | 0.006 | -0.0001 to 0.012  P = 0.062 | reference | | - | |
| PP | -0.092 | -0.100 to -0.083  P < 0.001 | -0.098 | -0.111 to -0.084  P < 0.001 | reference | |
| MAP | 0.011 | 0.007 to 0.015  P < 0.001 | 0.005 | 0.003 to 0.007  P = 0.002 | 0.103 | 0.091 to 0.115  P < 0.001 |
| **Stroke** | vs. SBP | | vs. DBP | | vs. PP | |
|  | difference  value | 95% CI  P value | difference  value | 95% CI  P value | difference  value | 95% CI  P value |
| SBP | reference | | - | | - | |
| DBP | 0.016 | 0.005 to 0.026  P = 0.002 | reference | | - | |
| PP | -0.106 | -0.119 to -0.092  P < 0.001 | -0.122 | -0.142 to -0.102  P < 0.001 | reference | |
| MAP | 0.019 | 0.012 to 0.024  P < 0.001 | 0.003 | -0.002 to 0.007  P = 0.358 | 0.125 | 0.106 to 0.141  P < 0.001 |
| **CAD** | vs. SBP | | vs. DBP | | vs. PP | |
|  | difference  value | 95% CI  P value | difference  value | 95% CI  P value | difference  value | 95% CI  P value |
| SBP | reference | | - | | - | |
| DBP | -0.001 | -0.008 to 0.007  P = 0.861 | reference | | - | |
| PP | -0.080 | -0.089 to -0.071  P < 0.001 | -0.079 | -0.093 to -0.065  P < 0.001 | reference | |
| MAP | 0.006 | 0.002 to 0.011  P = 0.018 | 0.007 | 0.004 to 0.010  P = 0.358 | 0.087 | 0.073 to 0.098  P < 0.001 |

Abbreviations: MACE, major adverse cardiovascular event; CAD, coronary arterial disease; SBP, systolic blood pressure; DBP, diastolic blood pressure; PP, pulse pressure; MAP, mean arterial pressure

Supplementary Table 10. Unadjusted hazard ratios and multivariate adjusted hazard ratios for MACE, stroke, CAD in participants aged over 50 years not receiving antihypertensive medications. (per SD)

| MACE | Crude | | Model 1 | | Model 2 | |
| --- | --- | --- | --- | --- | --- | --- |
|  | Hazard ratios (95% CI)  C-Statistics (95% CI) | *p* | Hazard ratios (95% CI)  C-Statistics (95% CI) | *p* | Hazard ratios (95% CI)  C-Statistics (95% CI) | *p* |
| SBP | 1.38 (1.33-1.43)  0.589 (0.566-0.614) | <0.001 | 1.32 (1.27-1.38)  0.625 (0.603-0.648) | <0.001 | 1.30 (1.25-1.35)  0.637 (0.614-0.660) | <0.001 |
| DBP | 1.37 (1.31-1.42)  0.589 (0.565-0.612) | <0.001 | 1.31 (1.26-1.37)  0.622 (0.600-0.644) | <0.001 | 1.29 (1.24-1.35)  0.634 (0.611-0.656) | <0.001 |
| PP | 1.19 (1.15-1.24)  0.541 (0.516-0.565) | <0.001 | 1.16 (1.11-1.21)  0.607 (0.585-0.629) | <0.001 | 1.15 (1.10-1.19)  0.624 (0.601-0.646) | <0.001 |
| MAP | 1.39 (1.34-1.44)  0.594 (0.570-0.617) | <0.001 | 1.34 (1.28-1.39)  0.626 (0.603-0.648) | <0.001 | 1.31 (1.26-1.37)  0.637 (0.614-0.660) | <0.001 |
| Stroke | Crude | | Model 1 | | Model 2 | |
|  | Hazard ratios (95% CI)  C-Statistics (95% CI) | *p* | Hazard ratios (95% CI)  C-Statistics (95% CI) | *p* | Hazard ratios (95% CI)  C-Statistics (95% CI) | *p* |
| SBP | 1.46 (1.37-1.56)  0.605 (0.562-0.647) | <0.001 | 1.43 (1.33-1.53)  0.625 (0.585-0.665) | <0.001 | 1.41 (1.32-1.51)  0.629 (0.588-0.669) | <0.001 |
| DBP | 1.49 (1.40-1.60)  0.613 (0.572-0.655) | <0.001 | 1.47 (1.37-1.58)  0.626 (0.586-0.666) | <0.001 | 1.45 (1.35-1.56)  0.629 (0.589-0.670) | <0.001 |
| PP | 1.20 (1.12-1.29)  0.543 (0.500-0.586) | <0.001 | 1.17 (1.09-1.26)  0.592 (0.552-0.632) | <0.001 | 1.16 (1.08-1.24)  0.602 (0.562-0.642) | <0.001 |
| MAP | 1.50 (1.41-1.60)  0.615 (0.573-0.657) | <0.001 | 1.48 (1.38-1.58)  0.629 (0.589-0.670) | <0.001 | 1.46 (1.36-1.57)  0.632 (0.592-0.673) | <0.001 |
| CAD | Crude | | Model 1 | | Model 2 | |
|  | Hazard ratios (95% CI)  C-Statistics (95% CI) | *p* | Hazard ratios (95% CI)  C-Statistics (95% CI) | *p* | Hazard ratios (95% CI)  C-Statistics (95% CI) | *p* |
| SBP | 1.32 (1.26-1.38)  0.578 (0.550-0.607) | <0.001 | 1.25 (1.19-1.32)  0.623 (0.597-0.649) | <0.001 | 1.23 (1.17-1.29)  0.6395 (0.6127-0.6663) | <0.001 |
| DBP | 1.29 (1.23-1.36)  0.574 (0.545-0.602) | <0.001 | 1.22 (1.16-1.29)  0.618 (0.592-0.645) | <0.001 | 1.20 (1.14-1.27)  0.636 (0.609-0.662) | <0.001 |
| PP | 1.18 (1.13-1.24)  0.538 (0.508-0.567) | <0.001 | 1.14 (1.09-1.20)  0.613 (0.587-0.639) | <0.001 | 1.13 (1.08-1.19)  0.632 (0.606-0.659) | <0.001 |
| MAP | 1.32 (1.26-1.38)  0.580 (0.551-0.608) | <0.001 | 1.25 (1.19-1.32)  0.622 (0.595-0.648) | <0.001 | 1.23 (1.17-1.29)  0.638 (0.611-0.665) | <0.001 |

Data are presented as hazard ratios (95% confidence intervals).

Model 1 was adjusted for gender, BMI, drinking habits, smoking status, and physical exercise.

Model 2 was adjusted for gender, BMI, drinking habits, smoking status, physical exercise, the presence of IFG, and the presence of dyslipidemia.

Abbreviations: SD, standard deviation; CI, confidence intervals; SBP, systolic blood pressure; DBP, diastolic blood pressure; PP, pulse pressure; MAP, mean arterial pressure; MACE, major adverse cardiovascular events; CAD, coronary artery disease; BMI, Body mass index; IFG, impaired fasting glucose.

Supplementary Table 11. Comparison of area under the curve of SBP, DBP, PP, and MAP with incident MACE, stroke, and CAD in participants aged over 50 years not receiving antihypertensive medications.

| **MACE** | vs. SBP | | vs. DBP | | vs. PP | |
| --- | --- | --- | --- | --- | --- | --- |
|  | difference  value | 95% CI  P value | difference  value | 95% CI  P value | difference  value | 95% CI  P value |
| SBP | reference | | - | | - | |
| DBP | -0.010 | -0.027 to 0.008  P = 0.282 | reference | | - | |
| PP | -0.048 | -0.071 to -0.025  P < 0.001 | -0.037 | -0.075 to -0.001  P = 0.044 | reference | |
| MAP | -0.013 | -0.002 to 0.010  P = 0.78 | 0.008 | 0.002 to 0.015  P = 0.014 | 0.046 | 0.014 to 0.078  P = 0.004 |
| **Stroke** | vs. SBP | | vs. DBP | | vs. PP | |
|  | difference  value | 95% CI  P value | difference  value | 95% CI  P value | difference  value | 95% CI  P value |
| SBP | reference | | - | | - | |
| DBP | 0.006 | -0.309 to 0.038  P = 0.774 | reference | | - | |
| PP | -0.068 | -0.096 to -0.040  P < 0.001 | -0.073 | -0.129 to -0.014  P = 0.01 | Reference | |
| MAP | 0.010 | -0.013 to 0.029  P = 0.37 | 0.005 | -0.008 to 0.020  P = 0.524 | 0.077 | 0.032 to 0.121  P = 0.002 |
| **CAD** | vs. SBP | | vs. DBP | | vs. PP | |
|  | difference  value | 95% CI  P value | difference  value | 95% CI  P value | difference  value | 95% CI  P value |
| SBP | reference | | - | | - | |
| DBP | -0.015 | -0.036 to 0.004  P = 0.124 | reference | | - | |
| PP | -0.036 | -0.066 to -0.008  P = 0.01 | -0.022 | -0.067 to 0.026  P = 0.324 | reference | |
| MAP | -0.006 | -0.006 to 0.007  P = 0.376 | 0.009 | 0.003 to 0.017  P = 0.004 | -0.010 | 0.031 to 0.070  P = 0.14 |

Abbreviations: MACE, major adverse cardiovascular event; CAD, coronary arterial disease; SBP, systolic blood pressure; DBP, diastolic blood pressure; PP, pulse pressure; MAP, mean arterial pressure

Supplementary Table 12. Baseline characteristics of Included versus Excluded participants not receiving antihypertensive medications

|  | Included | Excluded | P-value |
| --- | --- | --- | --- |
| N | 163,956 | 28,269 | - |
| Age (y) | 42.8 (9.0) | 43.4 (11.7) | <0.001 |
| Gender (males/females) (%) | 118,753/45,203 (72.4/27.6) | 13,328/14,941 (47.1/52.9) | <0.001 |
| Body mass index (kg/m^2^) | 22.8 (3.4) | 22.5 (3.8) | <0.001 |
| Systolic blood pressure (mmHg) | 117.7 (14.4) | 117.1 (15.9) | <0.001 |
| Diastolic blood pressure (mmHg) | 73.1 (10.9) | 72.2 (11.7) | <0.001 |
| Pulse pressure (mmHg) | 44.6 (8.8) | 44.8 (9.7) | <0.001 |
| Mean arterial pressure (mmHg) | 88.0 (11.5) | 87.0 (13.2) | <0.001 |
| Triglycerides (mg/dL) | 107.3 (88.5) | 99.3 (85.4) | <0.001 |
| HDL cholesterol (mg/dL) | 60.9 (15.3) | 63.1 (16.1) | <0.001 |
| LDL cholesterol (mg/dL) | 122.0 (31.7) | 117.0 (32.0) | <0.001 |
| Fasting plasma glucose (mg/dl) | 93.5 (16.3) | 94.4 (20.7) | <0.001 |
| Glucose-lowering medications (+/-) | 2,306/161,650 (1.4/98.6) | 868/26,469 (3.2/96.8) | <0.001 |
| Lipid-lowering medications (+/-) | 3,437/160,519 (2.1/97.9) | 1,394/25,939 (5.1/94.9) | <0.001 |
| Smoking (none/past/current)  (%) | 91,224/21,033/51,699  (55.6/12.8/31.5) | 15,971/3,954/7,407  (58.4/14.5/27.1) | <0.001 |
| Drinker (+/-) (%) | 36,030/127,926 (22.0/78.0) | 4,950/22,236 (18.2/81.8) | <0.001 |
| Physical exercise (+/-) (%) | 28,084/135,872 (17.1/82.9) | 5,089/22,161 (18.7/81.3) | <0.001 |

Data are presented as mean (standard deviation, or percentage) or absolute number.

Abbreviations: LDL, low-density lipoprotein; HDL, high-density lipoprotein.
